# Oxidized Phospholipids in Control of Endothelial Barrier Function: Mechanisms and Implication in Lung Injury

**DOI:** 10.3389/fendo.2021.794437

**Published:** 2021-11-23

**Authors:** Pratap Karki, Konstantin G. Birukov

**Affiliations:** ^1^ Department of Medicine, University of Maryland School of Medicine, Baltimore, MD, United States; ^2^ Department of Anesthesiology, University of Maryland School of Medicine, Baltimore, MD, United States

**Keywords:** oxidized phospholipids, OxPAPC, endothelial barrier, inflammation, lung injury, Rho GTPases, receptor

## Abstract

Earlier studies investigating the pathogenesis of chronic vascular inflammation associated with atherosclerosis described pro-inflammatory and vascular barrier disruptive effects of lipid oxidation products accumulated in the sites of vascular lesion and atherosclerotic plaque. However, accumulating evidence including studies from our group suggests potent barrier protective and anti-inflammatory properties of certain oxidized phospholipids (OxPLs) in the lung vascular endothelium. Among these OxPLs, oxidized 1-palmitoyl-2-arachdonyl-sn-glycero-3-phosphocholine (OxPAPC) causes sustained enhancement of lung endothelial cell (EC) basal barrier properties and protects against vascular permeability induced by a wide variety of agonists ranging from bacterial pathogens and their cell wall components, endotoxins, thrombin, mechanical insults, and inflammatory cytokines. On the other hand, truncated OxPLs cause acute endothelial barrier disruption and potentiate inflammation. It appears that multiple signaling mechanisms triggering cytoskeletal remodeling are involved in OxPLs-mediated regulation of EC barrier. The promising vascular barrier protective and anti-inflammatory properties exhibited by OxPAPC and its particular components that have been established in the cellular and animal models of sepsis and acute lung injury has prompted consideration of OxPAPC as a prototype therapeutic molecule. In this review, we will summarize signaling and cytoskeletal mechanisms involved in OxPLs-mediated damage, rescue, and restoration of endothelial barrier in various pathophysiological settings and discuss a future potential of OxPAPC in treating lung disorders associated with endothelial barrier dysfunction.

## Introduction

In various pathological conditions, especially during inflammation and oxidative stress, circulating and cell membrane phospholipids undergo oxidation to form a diverse group of oxidized phospholipids (OxPLs). These bioactive OxPLs possess profound biological activities and exert both beneficial and harmful effects on human body governed by their biochemical and biophysical properties. It has been long recognized that OxPLs accumulate in atherosclerotic lesions (1–3), and later studies have shown OxPLs elevation in other cardiopulmonary disorders driven by increased inflammatory responses [Reviewed in (4)]. However, emerging evidence suggests that OxPLs not only induce and propagate inflammatory response but may also contribute to the host response, resolution of inflammation and protection of vascular endothelial barrier properties (5). Endothelial cell (EC) lining of the vascular lumen forms a barrier that controls the passage of fluids, macromolecules, and immune cells between the blood and underlying tissue. The disruption of this selective and semi-permeable barrier results in uncontrolled passage of harmful substances leading to the development of pulmonary edema and acute lung injury (ALI) observed in many disorders: sepsis, severe infection, trauma, toxin inhalation, etc., and culminating in acute respiratory distress syndrome (ARDS) (6, 7). OxPLs play a dual role in regulating endothelial function: some species of OxPLs have been involved in enhancing endothelial barrier integrity while others increased endothelial permeability. The wide structural heterogeneity of OxPLs has been suggested to be responsible for their contrasting biological activities (8, 9). For instance, full length oxidation products of a major membrane phospholipid 1-palmitoyl-2-arachidonoyl-*sn*-glycero-3-phosphocholine (OxPAPC, focus of this review) possess potent endothelial barrier protective and anti-inflammatory properties (10). On the other hand, truncated or fragmented products of PAPC oxidation induce acute endothelial barrier disruption and inflammation (11). Multiple signaling pathways including receptor-mediated and cytoskeletal reorganization have been implicated in OxPAPC-induced upregulation of endothelial function which will be discussed in detail in the following sections.

## Generation of OxPLs

A diverse spectrum of OxPLs are generated from the oxidation of phospholipids that contain polyunsaturated fatty acids (PUFA) at their *sn*-2 position. PUFAs are highly prone to oxidative modifications by a group of specific enzymes such as cyclooxygenases (COX) and lipoxygenases (LOX) or reactive oxygen species (ROS)-mediated non-enzymatic lipid oxidation (9, 12, 13). Briefly, fatty acids such as arachidonic acid (AA) and linoleic acid (LA) are released from membrane phospholipids by phospholipase A2 (PLA2). In turn, COX-mediated oxidation of AA produces prostaglandins (PGs) and thromboxanes whereas LOX-catalyzed metabolic pathways yield leukotrienes, lipoxins, resolvins, protectins and eoxins. Multiple studies have shown the modulation of endothelial function by PGs but it is out of the scope of this manuscript. Briefly, PGI2, PGE2 and PGA2 exhibited potent, although transient barrier protective activities in pulmonary endothelium in comparison to OxPAPC effects (14, 15). These PGs enhanced endothelial barrier by stimulating cAMP production leading to PKA-dependent activation of Rac and PKA-independent activation of Epac/Rap1/Rac1 signaling cascade (14). PGE2, PGI2, and PGA2 also protect against thrombin-induced hyperpermeability *in vitro* and LPS-induced lung injury *in vivo* (15, 16). Similarly, PGD2 has been shown to enhance endothelial barrier function and protect against ALI (17–19). Furthermore, stable analogs of PGs such as beraprost and iloprost possess protective and anti-inflammatory activities in pulmonary endothelium (20–23). Both COX and LOX are involved in the generation of hydroxyeicosatetraenoic acids (HETEs) from AA oxidation and hydroxyoctadecadienoic acids (HODEs) from LA oxidation. Free radicals-mediated non-enzymatic oxidation of phospholipids produces a heterogenous mixture of bioactive OxPLs species through the classical pathway of lipid peroxidation characterized by initiation, propagation, and termination steps (24).

PAPC, a major membrane phospholipid, undergoes oxidation resulting in generation of wide varieties of full length as well as fragmented oxidized products. The full-length OxPAPC products contain same number of carbon atoms in oxidized arachidonic fatty acid chain as in their precursor. Examples of such products include 1-palmitoyl-2-(5, 6-epoxyisoprstane E2)-sn-glycero-3-phosphatidyl choline (PEIPC) and 1-palmitoyl-2-(5,6 epoxycyclopentenone) *sn* -glycero-3-phsphocholine (5,6-PECPC), among others. Conversely, fragmented OxPAPC products are oxidatively truncated at the *sn*-2 position. 1-Palmitoyl-2-(5-oxovaleroyl)-*sn*-glycero-phosphatidylchonine (POVPC), 1-palmitoyl-2glutaroyl *sn*-glycero-phosphocholine (PGPC), 1-(palmitoyl)-2-(5-keto-6-octene-dioyl)phosphatidylcholine (KOdiA-PC) represent examples of such products (25). Besides phosphatidylcholine, other phospholipids containing different polar groups such as 1-palmitoyl-2-arachidonoyl-sn-glycero-3-phosphatidylethanolamine (PAPE) and 1-palmitoyl-2-arachidonoyl-sn-glycero-3-phosphatidylserine (PAPS) are also subjected to oxidative modifications and exhibit similar effects on endothelial barrier regulation and inflammation (15). Furthermore, lipid peroxidation also results in the production of 4-hydroxy-2-nonenal (4-HNE) and malondialdehyde (MDA) (24).

## Contrasting Effects of OxPLs on Endothelial Function

**Figure 1:**
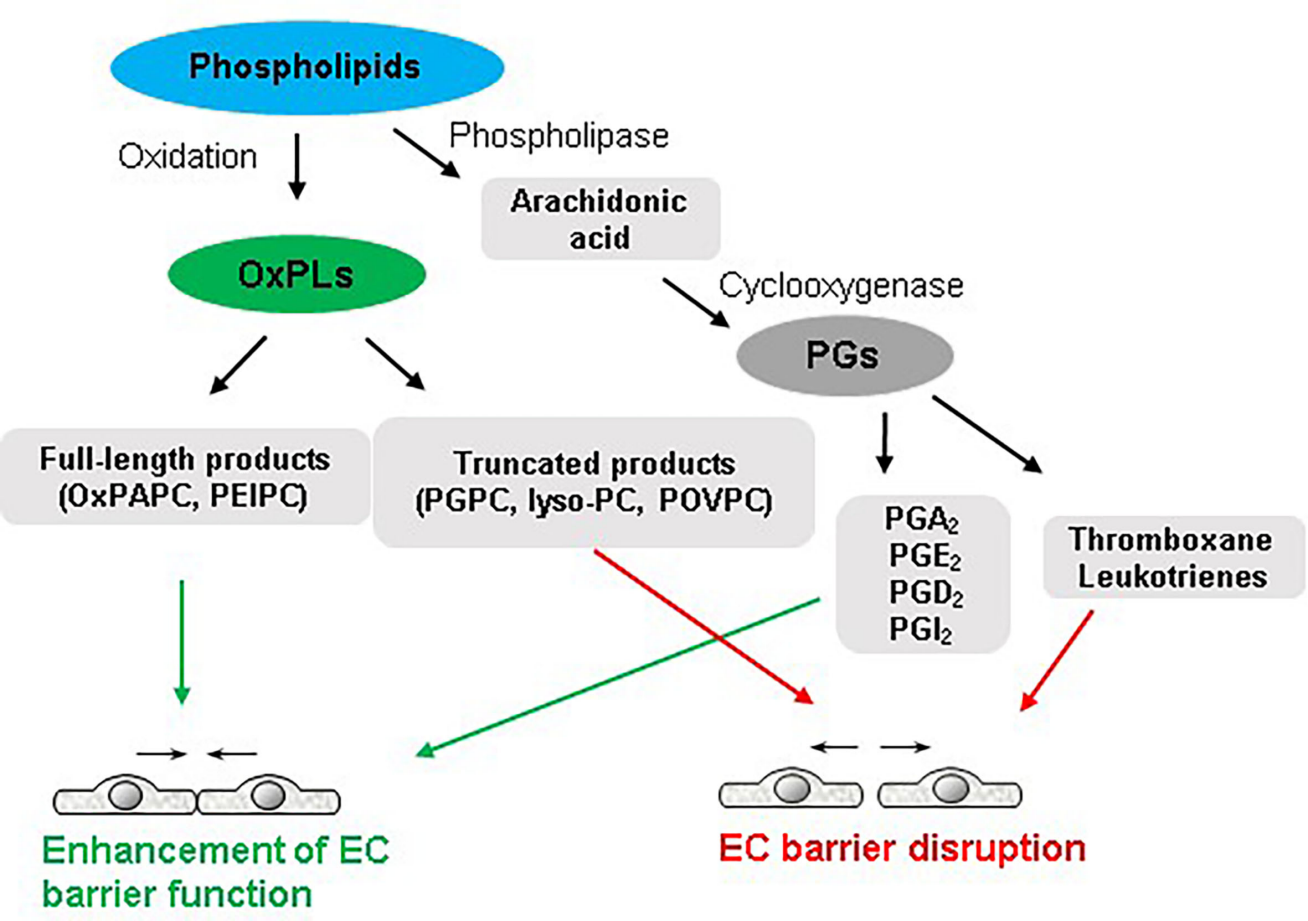
Dual role of phospholipids in endothelial function. Full-length oxidized products of phospholipids and some groups of PGs enhance endothelial barrier integrity. In turn, truncated OxPLs and some products of arachidonic acid such as thromboxane and leukotrienes disrupt endothelial barrier.

A continuous monolayer of EC covers vascular lumen and provides a highly selective semi-permeable barrier between the circulation and underlying tissues. EC barrier controls the passage of fluids, solutes, and cells across the vascular endothelium. Various injurious stimuli disrupt the endothelial barrier integrity leading to an increased endothelial permeability for macromolecules and immune cells that is a hallmark of numerous disorders such as pulmonary edema, ARDS, sepsis, and other pathologies (26–29). Bioactive lipid mediators may have both, positive and negative impact on endothelial barrier function (**Figure 1**). The principal reason behind the contrasting effects of various species of OxPLs is best explained by structure-function analysis. It appears that different molecular species present in OxPAPC govern its function on endothelial barrier. Precisely, full-length OxPLs species such as PEIPC and PECPC mediate barrier protective and barrier-enhancing effects of OxPAPC whereas sn-2-fragmented OxPLs such as PGPC and POVPC induce endothelial permeability (11, 30). Likewise, polar head groups present in OxPAPC also modulate its barrier function. OxPLs with negatively or positively charged polar head groups such as oxidized phosphocholine and phosphoserine exerted potent and sustained barrier-protective effects (31). But oxidized glycerophosphate lacking polar head group had only transient EC barrier protective effects (31).

A role of OxPLs in various cardiopulmonary disorders including ALI (32), ARDS (33), pulmonary hypertension (34), asthma (35), and cystic fibrosis (36) has been suggested by many studies showing the presence of elevated levels of lipid peroxidation products. Furthermore, OxPLs have a direct impact on EC function, as evidenced by OxPLs-driven effects in chronic vascular inflammation associated with atherosclerosis and manifested by enhanced adhesion of monocytes to EC, augmented expression of several inflammatory genes and secretion of inflammatory cytokines and chemokines such as interleukin-8 (IL-8) and monocyte chemotactic protein-1 (MCP-1) (2, 8, 37). OxPLs are also shown to cause pro-thrombotic phenotype of EC (38, 39) and act as potent oxidative stress inducers in EC (40, 41).

In contrast to these pathological roles of OxPLs, multiple studies have demonstrated potent anti-inflammatory and endothelial barrier protective functions of OxPLs in host defense against bacterial pathogens. Initial studies demonstrated a protective role of OxPAPC against endothelial dysfunction caused by bacterial wall lipopolysaccharide (LPS) in cultured EC as well as in murine models of ALI (30, 38, 39). Later studies have reported the involvement of OxPAPC in rescue and repair of endothelial function disrupted by various injurious factors: live and killed bacteria, components of bacterial wall, edemagenic agonists (thrombin), inflammatory cytokines (TNFα, IL-6), and pathologic mechanical forces: high amplitude cyclic stretch, disturbed flow [Reviewed in (42)]. Several signaling pathways mediating beneficial effects of OxPLs have been described in endothelium (14). Anti-inflammatory roles of OxPAPC have been summarized in our recent reviews (10, 42). This review will solely focus on OxPAPC-mediated endothelial barrier function.

## Mechanisms of OxPAPC-Induced Enhancement of Endothelial Barrier Function

Endothelial barrier is a dynamic structure that constantly undergoes remodeling in response to mechanical forces and various agonists that positively or negatively regulate barrier function. Altered expression of cell junction proteins, the assembly-disassembly dynamics of adherens junction (AJ - VE-cadherin, α, β, γ, and p120-catenins, nectin) and tight junction (TJ - claudin-5, ZO-1, 2, 3, afadin) protein complexes and reorganization of endothelial cytoskeleton in response to chemical and mechanical stimulation are the major determinants of endothelial barrier integrity. While barrier-disruptive insults (edemagenic agonists or high magnitude cyclic stretch) stimulate stress fiber formation, actomyosin contraction, disassembly of cell junction complexes leading to cell retraction and formation of inter-cellular gaps, barrier-enhancing agonists (OxPAPC, hepatocyte growth factor, sphingosine 1-phosphate) or physiologic mechanical forces (laminar flow, low magnitude cyclic stretch) stimulate enhancement of cortical cytoskeleton, assembly and cooperation of AJ and TJ protein complexes (43, 44). Remodeling of cell junctions and actomyosin cytoskeleton is precisely controlled by signaling protein kinases and small GTPases.

### Receptor-Mediated Pathways

A number of receptor-associated signaling pathways mediating OxPAPC actions on lung endothelium has been described. The earlier studies discovered important anti-inflammatory effects of OxPAPC *via* interference with toll-like receptors (TLRs) inflammatory signaling cascade activated by LPS (45, 46). Analysis of OxPAPC-mediated endothelial barrier enhancing mechanisms revealed Akt-dependent transactivation of sphingosine 1-phosphate receptor 1 (S1P1) *via* threonine phosphorylation in caveolin-enriched microdomain that stimulated Rac1- and Rap1-dependent pathways of peripheral F-actin and cell junction enhancement (47). The role of caveolin and S1P1 in mediating the protective effects of OxPAPC was further verified *in vivo* in VILI-induced lung injury where siRNA-mediated knockdown of caveolin or S1P1 abolished the OxPAPC-mediated protection of vascular leak (47). Further analysis elaborated that OxPAPC-induced activation of S1P1 is dependent on the binding of OxPAPC to HTJ-1, a co-factor of cell surface receptor GRP78 (48). siRNA-mediated depletion of HTJ-1 in EC abolished OxPAPC-induced cortical actin formation and knockdown of mouse HTJ-1 homologue suppressed the protective effects of OxPAPC against IL-6 or VILI-induced lung vascular leak (48). Prostaglandin E receptor-4 (EP4) also appears to be involved in endothelial barrier-enhancing responses of OxPAPC as recent study demonstrated that EP4 mediates sustained phase of OxPAPC-induced barrier protective effects (49). A selective role of EP4 in mediating OxPAPC effects was established by the findings that OxPAPC specifically increased EP4 mRNA expression levels in EC and inhibitors targeting EP4 but no other receptors such as EP1-3, PGI2, PGF2, PGD2, and thromboxane had no inhibitory effects on OxPAPC-induced enhancement of EC barrier (49). The role of EP4 in mediating the protective effects of OxPAPC was further established in murine model of ALI as OxPAPC-administered endothelial specific EP4 knockout mice failed to recover from LPS-induced vascular leak and inflammation (49). We recently reported that lipoxin A4 formyl peptide receptor-2(FPR2/ALX) is involved in OxPAPC-induced protection against endothelial permeability caused by TNF-α (50). The endogenous depletion of only FPR2 but no other FPR subtypes inhibited OxPAPC-mediated attenuation of TNF-α-induced increase in endothelial permeability and inflammation suggested the specific involvement of FPR2. Moreover, FPR2 specific inhibitor and FPR2 knockout mice showed reduced inhibition of LPS-induced ALI by OxPAPC further confirmed an essential role of FPR2 in mediating protective effects of OxPAPC (50). Interestingly, FPR2 depletion did not have any inhibitory effects on OxPAPC-induced protection against thrombin-caused EC permeability, suggesting that FPR2 dependent protective pathways also rely on the nature of agonist (e.g., acute vs. chronic effects) that requires further investigations.

### Rho GTPases-Mediated Cytoskeletal Remodeling

**Figure 2:**
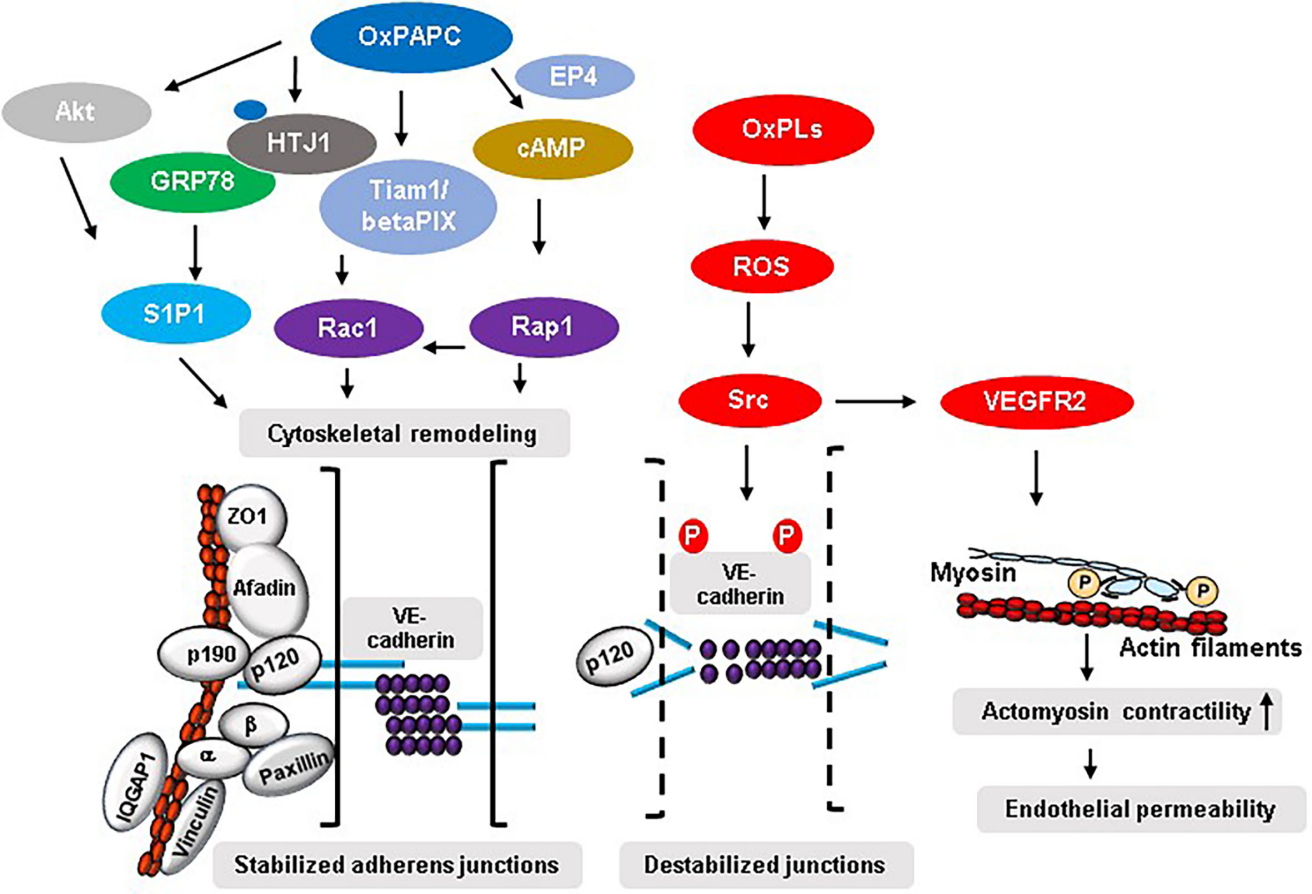
Mechanisms of OxPLs-mediated regulation of endothelial barrier. OxPAPC-induced activation of S1P1 in Akt or GRP78-dependent manner leads to an increased interaction between various AJ, TJ, and FA proteins resulting in enhancement of endothelial barrier integrity. Similarly, OxPAPC-mediated activation of Rac1 and Rap1 induces cytoskeletal reorganization favoring stabilized junctional assembly. On the other hand, truncated OxPLs-stimulated EC produce ROS that activates Src which phosphorylates tyrosine residues in VE-cadherin causing its internalization, degradation, and ultimately leading to disrupted endothelial barrier. Src also activates VEGFR2 that induces endothelial permeability *via* enhanced actomyosin contractility caused by increased myosin light chain phosphorylation.

Ras superfamily of small GTPases specifically Rho GTPases (RhoA, Rac1 and Cdc42) and Rap1 are critical regulators of endothelial barrier in physiological and pathological conditions (51). These GTPases control endothelial permeability by cycling between GTP-bound active and GDP-bound inactive states thereby driving the reorganization of EC junction-associated actin cytoskeleton (52, 53). Among the GTPases, RhoA mediates endothelial barrier disruption caused by various agents while Rac, Cdc42 and Rap1 are involved in maintaining and protecting EC barrier (44, 51, 52, 54). Our initial study showed that cytoskeletal remodeling driven by the combined activation of Rac1 and Cdc42 mediates OxPAPC-induced upregulation of endothelial barrier function (30). The follow-up study revealed that Rac/Cdc42-specific guanine nucleotide exchange factors (GEFs) Tiam1 and betaPIX are involved in Rac-mediated endothelial barrier protective effects of OxPAPC (55). The mechanistic analysis demonstrated that a novel interaction between focal adhesion (FA) and AJ complexes facilitated by the association of paxillin and beta-catenin is essential for Rac/Cdc42-dependent barrier protective responses of OxPAPC (56). Furthermore, Rac effector p21-activated kinase (PAK1)-mediated phosphorylation of paxillin serves as a positive-feedback regulatory pathway contributing to sustained enhancement of endothelial barrier by OxPAPC (57). A further evidence of crucial role of Rac signaling in mediating barrier protective effects of OxPAPC was substantiated by our recent study where knockdown of Rac1/Cdc42 effector IQGAP1 inhibited OxPAPC-induced enhancement of endothelial barrier by suppressing membrane accumulation of AJ proteins VE-cadherin and p120-catenin, and cortactin (58). Consistent with the role of RhoA in endothelial barrier disruption, Rac-mediated barrier protective effects of OxPAPC were achieved by the activation of p190RhoGAP, a negative regulator of RhoA (59). Briefly, OxPAPC-stimulated ECs showed tyrosine phosphorylation and peripheral translocation of p190RhoGAP leading to its association with AJ protein p120-catenin. More importantly, knockdown of p190RhoGAP abolished the protective effects of OxPAPC against the vascular leak induced by pathological cyclic stretch *in vitro* and ventilator-induced lung injury (VILI) *in vivo* (59). Later studies have shown that interaction of p120-catenin with p190RhoGAP was essential for the recruitment of the latter at cell periphery to inhibit Rho signaling (60). This notion was established from the findings that transient expression of p120-catenin mutant lacking 820-843 amino acids residues at C-terminal domain inhibited membrane translocation of p190RhoGAP causing an attenuation of OxPAPC-induced endothelial barrier enhancement resulted from the sustained activation of Rho signaling and suppression of Rac1 (60). In addition to Rac1/Cdc42, Rap1 is also involved in mediating the barrier protective signals of OxPAPC. Membrane accumulation and increased association of AJ proteins VE-cadherin, p120-catenin, and β-catenin and TJ proteins ZO-1, Occludin, and JAM-A were dependent on OxPAPC-induced Rap1 activation (61). An important role of Rap1 in OxPAPC-induced beneficial effects on vascular endothelium was further substantiated by the findings that Rap1 knockdown suppresses OxPAPC-derived enhancement of EC barrier and represses OxPAPC-induced protection against VILI (61). Our study also demonstrated an essential role of Afadin during Rap1-mediated barrier protective responses of OxPAPC (62). In this regard, OxPAPC treatment of ECs caused Rap1-dependent accumulation of Afadin at cell periphery and its increased association with AJ and TJ proteins- p120-catenin and ZO-1, respectively (62). Consistently, siRNA-mediated knockdown of Afadin or Rap1 binding-defective mutant of Afadin inhibited OxPAPC-induced enhancement of endothelial barrier and Afadin-depleted mice were no longer protected by OxPAPC against VILI-caused vascular leak. Lastly, one of our studies have suggested an existence of Rho GTPase-independent interaction of junction proteins that play a role in OxPAPC-induced positive regulation of endothelial barrier function. In this line, mechanosensitive adaptor protein vinculin seems to be an important regulator of OxPAPC-induced endothelial barrier protection *via* its interaction with AJ protein VE-cadherin (63). The direct activation of Rac1 and Rap1 by OxPAPC leading to enhanced assembly of endothelial cell junction complexes and cytoskeletal remodeling leading to strengthening of endothelial barrier are the best known two critical mechanisms of OxPAPC-induced positive regulation of endothelial barrier (**Figure 2**).

### Intracellular Signaling Pathways

A role of intracellular signaling pathways involving various kinases has also been described to mediate the barrier protective signals of OxPAPC. OxPAPC-stimulated ECs showed time-dependent activation of protein kinase A (PKA), protein kinase C (PKC), Raf-MEK MAP kinase cascade, and JNK MAP kinase (64). Furthermore, OxPAPC treatment also induced a transient increase in global tyrosine phosphorylation and Src kinase-dependent phosphorylation of FA proteins- paxillin and FA kinase in EC (64). By employing pathways-specific pharmacological inhibitors, our study showed that PKA, PKC, Src family kinases, and tyrosine kinases play essential roles during OxPAPC-induced enhancement of endothelial barrier (14). The same study ruled out the involvement of Rho kinase, PI3 kinase, and p38 MAP kinases in mediating Rac-mediated barrier enhancing responses of OxPAPC. OxPAPC has been shown to induce the expression of anti-inflammatory proteins such as heme oxygenase-1 *via* activation of PKA, PKC, and MAP kinase pathways (65), and *via* Nrf2 activation (66) but its impact on endothelial barrier function needs to be investigated. An elevation of cAMP levels *via* PKA-mediated or PKA-independent and Rap-mediated pathways induced by OxPAPC are also suggested to play roles in barrier protective effects of OxPAPC (14). **Figure 2** demonstrates a network of signaling pathways activated by OxPAPC and involved in EC barrier regulation. Of note, EC barrier protective effects of OxPAPC seems to vary among different EC types. In general, EC of microvascular origin tend to be more sensitive towards OxPAPC effects with more pronounced protective effects against agonist-induced permeability (67). It will be interesting to study if a variation of OxPAPC effects exists in the endothelium from different vascular beds such as lung, heart, and brain.

## Mechanisms of OxPLs-Induced Disruption of Endothelial Barrier

As discussed above, truncated products of phospholipids oxidation possess endothelial barrier disruptive properties. Moreover, even OxPAPC at higher concentration (≥ 50 µg/ml) causes barrier disruption as opposed to its barrier enhancing function at lower concentrations (5-20 µg/ml) (11, 68). These contrasting biological effects of OxPLs on lung endothelium are largely due to the different signaling pathways activated by them. This notion was best exemplified by one of our studies where the same protein vinculin associated with two distinct proteins- with FA protein talin during thrombin-induced barrier disruption and with VE-cadherin during OxPAPC-induced barrier enhancement (63). The selective activation of various signaling pathways depending on the dose and structural variations of OxPLs appears to be the primary determinants of their biological function.

### Role of Vascular Endothelial Growth Factor Receptor 2 (VEGFR2)

The binding of VEGFA to VEGFR2 results in the phosphorylation of the latter leading to the signaling cascades that regulate endothelial survival, proliferation, and migration but VEGFR2 activation is also linked to increased endothelial permeability (69, 70). Interestingly, OxPLs are shown to activate VEGFR2 in EC in Src-dependent manner (71). A role of VEGFR2 in OxPLs-induced endothelial barrier disruption was revealed by our study where only higher concentrations of OxPAPC increased endothelial permeability *via* VEGFR2 activation (72). Mechanistically, higher OxPAPC dose-induced endothelial barrier disruption was accompanied by Rho activation-led increased actomyosin contractility evidenced by increased phosphorylation of myosin phosphatase (MYPT1) and myosin light chain (MLC) that were attenuated by siRNA-mediated knockdown of VEGFR2 (72).

### Reactive Oxygen Species (ROS)-Mediated VE-Cadherin Phosphorylation

VE-cadherin is the major adhesive protein present in human EC that is critically essential for maintenance of endothelial barrier integrity (73). Tyrosine phosphorylation of this AJ protein leads to its disassociation from the complex with catenin proteins resulting in increased endothelial permeability (74). ECs treated with higher doses of OxPAPC showed the increased tyrosine phosphorylation of VE-cadherin at tyrosine-658 and tyrosine-731 that was absent in cells treated with low, barrier enhancing doses of OxPAPC (68). The endothelial barrier disruption induced by higher doses of OxPAPC was associated with increased protein tyrosine phosphorylation, activation of Src with phosphorylation at tyrosine 418 and dissociation of VE-cadherin complex with p120- and β-catenin. Furthermore, only high doses of OxPAPC exceeding 50 µg/ml exposure to EC resulted in elevation of ROS levels and anti-oxidant N-acetyl cysteine rescued endothelial barrier disruption caused by OxPAPC (68). An identical pathway of ROS production, Src activation, and VE-cadherin phosphorylation at tyrosine 658 and 731 was observed in ECs treated with PGPC, a truncated OxPLs product (11). Likewise, particulate matter-induced endothelial barrier disruption that is associated with increased production of truncated OxPLs involves similar mechanisms of increased oxidative stress-mediated tyrosine phosphorylation of VE-cadherin (75). Thus, ROS-mediated activation of Src followed by tyrosine phosphorylation of VE-cadherin leading to its internalization and degradation appears to be a unifying mechanism of OxPLs-induced endothelial barrier disruption.

## OxPLs in Endothelial Dysfunction-Derived Lung Diseases

It is now widely accepted that OxPLs play dual roles on regulating the function of vascular endothelium and thus they can be either potentially developed into therapeutics or utilized as targets of therapeutic interventions for various cardiopulmonary diseases that are developed secondary to endothelial dysfunction. In particular, with its proven endothelial barrier-protective and anti-inflammatory activities against a broad spectrum of injurious insults such as bacterial pathogens (76), thrombin (30), LPS (45, 77, 78), and mechanical forces represented by VILI or cyclic stretch (67), OxPAPC holds a strong therapeutic potential against these agonists-induced ALI, ARDS and sepsis. The central role of Rac/Rap1-mediated cytoskeletal reorganization in OxPAPC-induced upregulation of endothelial barrier function makes this molecule attractive therapeutic candidate since the activation of these GTPases act as a common platform for conveying barrier enhancing signals originating from numerous barrier protective agents. The involvement of a number of receptors and kinases in mediating the beneficial actions of OxPAPC on the lung endothelium further provides the opportunity to consider these multiple molecules and associated pathways for the therapeutics.

Some OxPLs products are detected at the site of tissue injury, inflammation and their deleterious effects on vascular endothelium has been well documented (2, 4). More recent studies suggest that these groups of OxPLs can also act as secondary injurious insults and exacerbate endothelial dysfunction. For instance, EC or mice lungs exposed to particulate matter from polluted air resulted in the generation of truncated OxPLs species such as POVPC, PGPC, and lyso-PC that caused acute endothelial barrier disruption (75). Furthermore, when combined with suboptimal dose of particulate matter that does not cause endothelial barrier dysfunction on its own, OxPLs augmented endothelial permeability, indicating their additive role in exacerbation of endothelial function in pre-existing disease conditions. This phenomenon was further evident with the presence of higher basal level of truncated OxPLs in aged mice and corresponding enhanced increase and delayed clearance of these phospholipids in LPS-treated aged mice compared to their younger counterparts (79). Aged mice were more susceptible to TNF-α-induced lung injury and non-toxic dose of POVPC when combined with TNF-α caused similar levels of lung inflammation. These observations suggest that generation of truncated OxPLs and their additive harmful effects with inflammatory agents further exacerbate lung injury/inflammation in aged population who may already have impaired anti-oxidant system. The definite role of truncated OxPLs as secondary injurious agonist was established by the selective removal of these lipids with platelet-activating factor acetyl hydrolase 2 (PAFAH2) which specifically hydrolyzes truncated OxPLs. The overexpression of PAFAH2 in EC rescued particulate matter- or inflammatory cytokines-induced endothelial barrier disruption, while pharmacological inhibition of PAFAH2 worsened lung injury in TNF-α-challenged mice (75, 79). A few other studies have also reported the presence of higher levels of truncated OxPLs in aged mice (80, 81), and increased production of fragmented phosphatidylcholine in human blood plasma as well as higher levels of ester-linked but lower level of ether-linked phosphatidylcholine in aged human individuals (82, 83). The changes in the levels of lipid profile of ester- vs ether-linked with aging has been attributed as a potential risk factor for the development of various diseases in elderly population. Specifically, ether-linked phospholipids are known to possess anti-oxidant activity and decreased serum levels of these PLs is linked to type 2 diabetes and hypertension (84, 85). Likewise, the oxidation of mitochondrial phospholipid cardiolipin has been shown to contribute to EC necrotic death and increased permeability (86), and hyperoxic lung injury (87). The profile of cardiolipin also undergoes changes with ageing as evidenced by the decrease of total cardiolipin content in mitochondria accompanied by an increase in oxidized forms in heart and brain from aged rats (88, 89). All of these findings suggest that altered lipid profile caused by excessive accumulation of bioactive truncated OxPLs due to exaggerated oxidative stress during aging may play a critical role in propagating associated adverse pathologies. On a positive note, targeting such lipid program switch to selectively inhibit the production or remove pre-formed harmful OxPLs products may provide a potential therapeutic avenue.

## Future Directions of OxPL Research and Clinical Applications

Although OxPLs oxidized *in vitro* are increasingly recognized for their anti-inflammatory and lung barrier-protecting activities *in vitro* and *in vivo*, they have a number of serious limitations precluding their therapeutic use. PAPC oxidation *in vitro* yields a complex, structurally diverse mixture of OxPLs. Such natural OxPLs are ineffective for *in vivo* therapy due to their various shortcomings. First, natural OxPLs with either barrier-protective or barrier-disruptive properties are rapidly degraded by phospholipases A that are very abundant in blood plasma, tissues, and cells. Second, natural OxPL mixtures contain a large proportion of oxidatively fragmented molecular species which demonstrate low anti-inflammatory activity in combination with increased toxicity and unwanted effects such as disruption of lung endothelial barrier. Therefore, engineering and synthesis of novel class of phospholipase-resistant phospholipids that have enhanced stability and better biological activities could lead to the discovery of effective drugs that preserve and protect endothelial function and ultimately can be used in the prevention and treatment of endothelial dysfunction-driven diseases. To overcome these limitations, we designed, synthesized, and experimentally tested biological properties of a phospholipase resistant phospholipid incorporating a prostaglandin I_2_ stable analog iloprost (ILO) as a prototype synthetic phospholipid compound with barrier-protective and anti-inflammatory properties (90). Such incorporation of ILO into synthetic phospholipid backbone led to more prolonged EC barrier enhancement and more pronounced anti-inflammatory effect, credited to its ability to cause a prolonged activation of Rap1 and Rac. In our continued efforts to synthesize such optimal phospholipids derivatives, recently an alkyl-amide OxPLs was synthesized that retained endothelial barrier enhancement and anti-inflammatory effects (91). As demonstrated by these examples, chemically modified phospholipase-resistant phospholipids may exhibit simultaneously two types of activities. First, these compounds inhibit signaling induced by TLRs (92). Second, these synthetic compounds enhance vascular endothelial barrier, reverse action of edemagenic mediators and prevent formation of lung edema *in vivo* (90, 91). Such poly-pharmacological mode of action can make these compounds especially effective for treatment of severe infections leading to the development of lung edema.

The generation of chemically diverse species of OxPLs and corresponding contrasting biological effects requires precise structure-function analysis of these lipid mediators to define their accurate pathophysiological roles. The advancements in liquid chromatography-mass spectrometry (LC-MS) techniques along with the use of sophisticated bioinformatics analysis has now made possible to detect minor modifications in OxPLs structure and its possible impact on biological activities. These advanced analysis and detection techniques combined with omics analysis approaches including lipidomics, metabolomic, transcriptomic, and proteomics will reveal the signaling cascades associated with OxPLs that will assist in identifying potential therapeutic targets for OxPLs-derived diseases. Furthermore, oxidative phospholipidomics analysis may serve as a valuable tool for identification of biomarkers as suggested by a recent study characterizing oxygenated cardiolipins and phosphatidylethanolamines as predictive biomarkers of apoptotic and ferroptotic cell death, respectively (93). Future projects also should consider exploring the role of OxPLs in the pathogenesis of other diseases besides its established role in cardiopulmonary disorders since recent findings suggest their involvement in other diseases including aging (79) and traumatic brain injury (94). The translation of potent beneficial effects of OxPAPC into clinics is the most exciting challenge ahead and other additional molecular targets such as receptors and Rho GTPases that mediate OxPAPC actions on lung endothelium could also be considered for therapeutic targets. Moreover, enzymes such as PAFAH2 represent another category of potential therapeutic candidates to prevent excessive accumulation of deleterious OxPL products.

## Conclusion

It is now widely appreciated that the complexity of contrasting biological effects of OxPLs largely relies on their structural heterogeneity with full-length oxidized products exerting beneficial effects and truncated species acting as pathogenic factor in various cardiopulmonary disorders. The studies so far have established that OxPAPC itself or signaling intermediates that mediate its functions on endothelium, barrier-disruptive OxPLs targeting enzymes such as PAFAH2, and structure-based synthetic analogues of barrier-protective OxPLs could be developed into therapeutics against acute endothelial dysfunction associated with injury, infection, or sepsis. The utilization of LC-MS advanced detection techniques in combination with next generation omics analytical tools has enabled to precisely monitor the lipid modifications and possibly use these modifications as biomarkers for various pathologies. Thus, the dual biological nature of OxPLs presents the opportunity to consider both “good” and “bad” oxidized products of circulating or membrane phospholipids for therapeutic interventions.

## Author Contributions

PK and KB outlined the review. PK drafted the manuscript and KB finalized. All authors contributed to the article and approved the submitted version.

## Funding

The studies in authors laboratory have been supported by RO1HL076259, R01HL146829, and RO1HL087823 grants from National Heart, Lung, and Blood Institute and RO1GM122940 from National Institute of General Medical Sciences to KB.

## Conflict of Interest

The authors declare that the research was conducted in the absence of any commercial or financial relationships that could be construed as a potential conflict of interest.

## Publisher’s Note

All claims expressed in this article are solely those of the authors and do not necessarily represent those of their affiliated organizations, or those of the publisher, the editors and the reviewers. Any product that may be evaluated in this article, or claim that may be made by its manufacturer, is not guaranteed or endorsed by the publisher.

## Abbreviations

AJ, adherens junction; ALI, acute lung injury; ARDS, acute respiratory distress syndrome; COX, cyclooxygenases; EC, endothelial cell; FA, focal adhesion; LOX, lipoxygenases; LPS, bacterial lipopolysaccharide; MLC, myosin light chain; MYPT1, myosin phosphatase 1; OxPLs, oxidized phospholipids; OxPAPC, oxidized 1-palmitoyl-2-arachidonoyl-*sn*-glycero-3-phosphorylcholine; PGs, prostaglandins; ROS, reactive oxygen species; S1P, sphingosine 1-phosphate; TLR, toll-like receptor; TJ, tight junction; TNF-α, tumor necrosis factor-α; VEGF, vascular endothelial growth factor; VILI, ventilator-induced lung injury.
